# Effects of State Medicaid Expansions Affecting Parental Eligibility on Children’s Healthcare Access and Health During the COVID-19 Pandemic

**DOI:** 10.1007/s10995-026-04292-x

**Published:** 2026-08-07

**Authors:** Joel M. James, George L. Wehby

**Affiliations:** 1 Department of Health Management and Policy, University of Iowa, Iowa City, IA USA; 2 Department of Economics, University of Iowa, Iowa City, IA USA; 3 National Bureau of Economic Research, Cambridge, MA USA; 4 Department of Health Management and Policy, The University of Iowa, 145 N. Riverside Dr., 100 College of Public Health Bldg., Room N232A, Iowa City, IA 52242-2007 USA

**Keywords:** State medicaid expansions, Healthcare access and utilization, Child health, Parent health

## Abstract

**Objective:**

To examine whether pre-pandemic state Medicaid expansions for adults affected children's healthcare access and utilization, child health, and parental health during the COVID-19 pandemic.

**Methods:**

The sample includes children from households with incomes up to 138% of the Federal Poverty Level using data from the 2016-2023 National Survey of Children’s Health. Child outcomes include insurance coverage, usual place for sick care, personal doctor or nurse, unmet healthcare needs, past 12-month utilization of any medical services, preventive medical and dental visits, mental health professional treatments, non-mental health specialist visits, emergency department visits, hospitalizations, and general/oral health ratings. Parent outcomes include physical and mental health ratings. Difference-in-differences models estimate year-by-year differences in outcomes between Medicaid expansion and non-expansion states.

**Results:**

There is a decline in usual place for sick care, personal doctor or nurse, and preventive medical visits in the first or second pandemic year and in child health in 2020/2023 in expansion versus non-expansion states. In contrast, there is an increase in mental health professional treatments and other specialist visits in 2021–2023 in expansion states. Lastly, there are declines in maternal physical/mental health and father’s mental health in 2020 but increase in father’s mental/physical health in 2023 in expansion states.

**Conclusion:**

During the pandemic, children in low-income households had less care continuity and preventive care but more mental health treatments and non-mental specialist visits in Medicaid expansion versus non-expansion states. There were also declines in both child and parent health. Findings potentially reflect intertwined effects from greater parental coverage but more pandemic-related social mobility restrictions disrupting routine/preventive care and worsening mental health more in expansion states.

**Supplementary Information:**

The online version contains supplementary material available at 10.1007/s10995-026-04292-x.

## Significance

What is already known on this subject?

What this study adds?

The COVID-19 pandemic dramatically distorted labor markets and health care access. How prior Affordable Care Act (ACA) Medicaid expansions affected children’s coverage, access to care, and health and parental health during the pandemic is not well known.

During the pandemic, children in low-income households had less care continuity and preventive care but more mental health treatments and non-mental specialist visits in states with prior ACA Medicaid expansions versus non-expansion states. There were also declines in both child and parent health. Findings potentially reflect intertwined effects from greater parental coverage but more pandemic-related social mobility restrictions in expansion states.

**Supplementary Information:**

The online version contains supplementary material available at 10.1007/s10995-026-04292-x.

## Introduction

The COVID-19 pandemic dramatically distorted labor markets and employment particularly for low-income workers. Many families faced job loss, reduced working hours, and lower earnings (Chen et al., 2022). Over 40% of families from low-income and lower-middle class households lost pay or income. These adverse economic impacts began shortly following the pandemic start and although differed across families they continued into the second year of the pandemic for many families. The unemployment rate among families nearly doubled in 2020 (9.8% compared to 4.9% in 2019), before declining in 2021 (6.7%), but remaining higher than pre-pandemic levels and returning close to pre-pandemic levels in 2022 (U.S. Bureau of Labor Statistics, 2023).

Research has shown that the Affordable Care Act (ACA) Medicaid expansions increased Medicaid coverage rates and improved access to health services and health status for low-income adults prior to the COVID-19 pandemic (Lyu et al., 2020; Lyu & Wehby, 2018, 2019; Miller & Wherry, 2019; Semprini et al., 2022). Some research has also shown that those prior Medicaid expansions provided health insurance protections for low-income adults during the pandemic, including less reporting of being uninsured, unpaid medical bills, unmet medical needs, and avoiding care due to cost (Auty et al., 2023; Benitez et al., 2023). Other work suggests potential benefits to mental health from these expansions during the pandemic (Mukhopadhyaya, 2022; Oyeka & Wehby, 2023). However, whether these Medicaid expansion protections for adults, including parents had spillover effects on children during the pandemic is not known.

This study examines whether the pre-pandemic ACA state Medicaid expansions for adults affected child healthcare access and utilization, child health, and parental health during the pandemic in low-income families. Evidence prior to the pandemic shows that increased parental Medicaid eligibility under the ACA improved parental health and access to care (Andreyeva et al., 2025; Gopalan et al., 2022; Lyu & Wehby, 2021; Margerison et al., 2020), but also increased children’s enrollment in Medicaid despite no changes in children’s own eligibility (Hudson & Moriya, 2017; Ugwi et al., 2019), suggesting positive spillover effects from parental to children’s Medicaid coverage. There is also evidence from before the pandemic of declines in maternal and infant mortality, suggesting positive spillover effects to infant health (Eliason, 2020) from those Medicaid expansions (Constantin & Wehby, 2023). In contrast, there is little evidence of direct improvements in children’s access to healthcare prior to the pandemic from those ACA Medicaid expansions (Dalziel et al., 2026; Gopalan et al., 2022), despite prior research showing higher likelihood for children in low-income families to complete well visits in states with higher Medicaid parental eligibility prior to the ACA Medicaid expansion (Venkataramani et al., 2017).

Importantly for this study, little is known about whether and how the pre-pandemic ACA Medicaid eligibility expansions for adults affected children’s coverage, access to care, and health, as well as parental health during the pandemic. Understanding these effects specifically during the pandemic is relevant for multiple reasons. As mentioned above, the pandemic resulted in a major employment shock that impacted many families and increased reliance on Medicaid. Indeed, the number of adults and children enrolled in Medicaid increased by 15.6 million (from 34.3 million) and 7 million (from 35.3 million), respectively, between February 2020 and April 2023 (Kaiser Family Foundation, 2026). Also contributing to this increase in enrollment is the continued eligibility of Medicaid beneficiaries under the 2020 Families First Coronavirus Response Act (FFCRA) which prohibited states from redetermining eligibility between March 2020 and March 2023. Consequently, adults and children who were enrolled in Medicaid at any time in this period continued to be enrolled even if they did not subsequently meet income eligibility criteria after initial enrollment. As such, even though the FFCRA did not modify income eligibility limits for initial enrollment, it provided continuous coverage for enrollees throughout this period. This continuous coverage may modify and plausibly intensify the effects of prior ACA Medicaid eligibility expansions on coverage and access during the pandemic since Medicaid expansion states have higher proportions of eligible families. Evidence suggests that the FFCRA Medicaid continuity provision increased Medicaid coverage and improved certain access measures for both adults (delaying care due to cost or needing but not affording care) and children (unmet care needs and having a usual place for sick care) although no discernable change in utilization outcomes were observed (Lyu & Wehby, 2024, 2026). For children, there is also improvement in parent/caregiver rating of the child’s health status (Lyu & Wehby, 2026). However, this evidence does not separate expansion and non-expansion states and therefore does not clearly address the question whether the FFCRA boosted the effects of prior ACA Medicaid expansions or not during the pandemic.

At the same time, the pandemic brought major disruptions to healthcare delivery and access that might also have modified the effects of prior ACA Medicaid expansions on parents' and children’s coverage and access to care. At a time of restrictions on social mobility and the de-emphasis of preventive care and discretionary or less urgent services from both the supply and demand side, evidence points to a meaningful decline in health services access and utilization during the pandemic for both adults and children (Lyu & Wehby, 2022, 2023a; Wright et al., 2021) and in preventive and discretionary care early during the pandemic (Whaley et al., 2020). Conceptually, this may attenuate some of the benefits of higher coverage rates in expansion states if covered families face access restrictions, especially for services deemed to be routine, preventive, or less urgent care. In contrast, families in expansion states might still have had greater access to other services such as specialized or mental healthcare. Moreover, the pandemic disrupted social networks, school activities, and other daily routines for parents and children and had significant adverse effects on family health and well-being, including worsening mental health among parents, increased emotional and behavioral problems among children, and declines in children’s flourishing and physical activity (Czeisler et al., 2021; Dudovitz et al., 2022; Lyu & Wehby, 2023b; Radhakrishnan et al., 2022a, 2022b; Suarez et al., 2025). With an elevated need for mental healthcare, insurance coverage becomes more salient for accessing and utilizing mental health services, which might amplify the benefits of ACA Medicaid expansions for low-income families during the pandemic.

## Methods

### Data and Sample

Data were obtained from the National Survey of Children’s Health (NSCH), a nationally representative cross-sectional survey conducted annually by the U.S. Census Bureau. The NSCH covers all 50 states, and the District of Columbia and collects data on children’s health, healthcare access and utilization, and household sociodemographic factors (Child and Adolescent Health Measurement Initiative, n.d.). The survey is completed by parents or other primary caregivers online, by mail, or by phone. The survey samples children aged 0-17 and is administered during the second half of each year (with some surveys completed by January of the following year). We include data from each year between 2016 and 2023 to examine both pre-pandemic trends and outcome changes during the pandemic. Data on state-level covariates come from the University of Kentucky Center for Poverty Research (UKCPR) National Welfare Data (UKCPR, 2025).

We limit the sample to children in households with incomes up to 138% of the federal poverty level (FPL) to focus on parentseligible for Medicaid based on income under the ACA expansions. Also, because we focus on spillover effects on children from parental coverage and to improve accuracy of responses, we include children whose parents were the survey respondents (83.7% of surveys). Moreover, since we focus on pre-pandemic state Medicaid expansions and aim to ensure the same sample composition of states in the treatment and control groups throughout the study period, we include 32 states that had expanded Medicaid under the ACA by 2016 in the treatment group and 10 states that had not expanded Medicaid by 2023 in the control group. For this reason, we exclude 7 states that expanded Medicaid in 2017–2023. Appendix Table A1 lists the included and excluded states by Medicaid expansion status. Depending on the outcome, the analytical sample ranges between 34,494 and 43,394 children with complete data for the regression models described below.

**Table 1:** Outcome descriptive statistics in 2016–2019 and 2020–2023 by medicaid expansion versus non-expansion status

	2016–2019			2020–2023		
	Expansion states	Non-expansion states	*p*-value	Expansion states	Non-expansion states	*p*-value
Health services access						
Covered by government assistance plan	82.0%	83.6%	0.171	82.6%	82.9%	0.7698
Any health insurance coverage	92.3%	85.7%	0.000***	90.1%	85.0%	0.000***
Has usual place for sick care	68.6%	67.6%	0.547	60.1%	61.6%	0.309
Personal Doctor/Nurse	61.7%	60.1%	0.332	57.2%	56.5%	0.604
Unmet healthcare needs	4.5%	6.2%	0.033*	5.2%	5.6%	0.563
Health services utilization in last 12 months						
Medical care utilization	75.2%	74.2%	0.516	72.8%	74.6%	0.157
Preventive medical visits	71.3%	70.6%	0.658	67.9%	70.3%	0.082
Preventive dental visits	71.1%	72.2%	0.512	83.8%	84.3%	0.690
Mental health professional	9.0%	9.4%	0.595	10.4%	8.7%	0.047
Non-mental health specialist visit	11.1%	11.2%	0.948	8.5%	8.8%	0.752
Emergency dept. visits	26.8%	29.4%	0.083	21.0%	24.3%	0.004**
Hospital stay	5.3%	5.6%	0.747	4.1%	3.2%	0.095
Child health outcomes						
General health	4.3	4.3	0.091	4.3	4.3	0.396
	(0.8)	(0.9)		(0.8)	(0.9)	
Oral health	3.9	3.9	0.417	3.8	3.9	0.288
	(1.0)	(1.1)		(1.1)	(1.0)	
Parent health outcomes						
Mother’s physical health	3.7	3.6	0.341	3.6	3.6	0.900
	(1.0)	(1.0)		(1.0)	(1.0)	
Mother’s mental health	3.9	4.0	0.283	3.8	3.8	0.241
	(1.0)	(1.0)		(1.1)	(1.0)	
Father’s physical health	3.7	3.8	0.040*	3.8	3.8	0.819
	(1.0)	(1.1)		(1.1)	(1.0)	
Father’s mental health	4.1	4.1	0.346	4.0	4.1	0.227
	(1.0)	(1.0)		(1.0)	(0.9)	
N	12,543	4704		20,653	7383	

All descriptive statistics are weighted by NSCH sampling weights. Means and standard deviations (in parentheses) are given for continuous and 1–5 categorical variables (where 1 indicates the lowest value and 5 the highest), while percents are given for binary outcomes. The table shows the significance of differences in those outcomes between expansion and non-expansion states within each period (based on bivariate chi-square tests for categorical variables and t-tests for continuous variables). Note that those differences in levels within each period do not represent the unadjusted version of the difference-in-differences event study estimates represented below as they only test differences in the levels of those outcomes between expansion and non-expansion states within each period

^*^This table shows descriptive statistics for the sample. Unadjusted frequencies for outcomes for the pre (2016–19) and post (2020–23) periods are given and sorted by Medicaid expansion and non-expansion status. Analytical sample size for each outcome, which includes observations with complete data on the outcome and all the covariates ranges between 29,157 and 36,768

^*^P < . 05, ^**^P < . 01, ^***^P < . 001

We consider two alternate samples in sensitivity analyses. The first analysis excludes Montana and Louisiana which expanded Medicaid in 2016 (the first year of the study period) considering that these states may have different outcome trends before the pandemic due to the immediate effects of their Medicaid expansions. The second analysis limits the sample to children with household income up to 100% of the FPL, considering that the ACA provided subsidies for marketplace coverage for households with income above 100% FPL that were also increased beginning in 2021 under the American Rescue Plan.

### Outcomes

The study evaluates children’s access to healthcare, healthcare utilization, and health status. Access outcome measures include binary (yes/no) indicators for whether the child: 1) is covered by a government assistance plan; 2) has any health insurance coverage; 3) has usual place for sick care; 4) has a personal doctor or nurse; and 5) has unmet healthcare needs. Healthcare utilization over the past 12 months (from the survey time) is measured by the following binary indicators for any: 1) medical services utilization 2) preventive medical visits; 3) preventive dental visits; 4) mental health professional treatments; 5) non-mental health specialist visits; 6) emergency department (ED) visits; and 7) hospital stays (first measured in 2018). The NSCH question about receiving treatment or counseling from a mental health professional, specifies that “mental health professionals include psychiatrists, psychologists, psychiatric nurses, and clinical social workers” (Child and Adolescent Health Measurement Initiative, n.d.). Also, the NSCH questionnaire describes specialists other than mental health professionals as “doctors like surgeons, heart doctors, allergy doctors, skin doctors, and others who specialize in one area of healthcare.” The questionnaire does not inquire further about the types of mental health services and specialist visits (U.S. Census Bureau, n.d.).

Health status is measured by the parent’s rating of the child’s general health on a five-category scale (poor, fair, good, very good, excellent), which we evaluate as a five-point numeric measure from 1 (poor) to 5 (excellent). Another five-category parent-rated measure is included for the child’s oral health status. In addition to these children’s outcomes, we also evaluate two measures of parent health as outcomes self-reported on a five-category (from poor to excellent) scale for mental health and physical health separately for mothers and fathers.

### Statistical Analysis

We estimate effects of the pre-pandemic Medicaid expansions on the study outcomes using an event study difference-in-differences (DD) regression model that compares year-by-year pre-post (before versus during the COVID-19 pandemic) differences in outcomes between ACA Medicaid expansion and non-expansion states. The model also evaluates pre-pandemic trends in these outcomes between the two state groups. The regression model is specified as follows:

$$ \begin{aligned} Y_{{ist}} = & \beta _{0} + \mathop \sum \limits_{{\begin{array}{*{20}c} {t = 2016} \\ {ref = 2019} \\ \end{array} }}^{{2023}} \beta _{t} (E_{s} \times \user2{T}_{t} ) \\ & \quad + \user2{X}_{{ist}} + \user2{C}_{{st}} + \user2{S}_{s} + \user2{T}_{t} + e_{{ist}} \\ \end{aligned} $$

*Y* represents one of the outcomes for child or parent i living in state *s* in survey year *t*. *E* is a binary (0/1) indicator for whether the state had expanded Medicaid by 2016 (*E* = 1) or not expanded by 2023 (*E* = 0); as noted above, states that expanded between 2016 and 2023 are excluded. **T ** includes fixed effects for survey years with 2019 as the reference year, which capture national trends in outcomes shared across states. *β*_*t*_ are the event-study difference-in-differences parameters. Compared to 2019, *β*_*2020,*_* β*_*2021,*_* β*_*2022*_ and *β*_*2023*_ estimate the pre-pandemic Medicaid expansion effects on the study outcomes during the pandemic years. In contrast, *β*_*2016,*_* β*_*2017,*_ and *β*_*2018*_ estimate pre-pandemic trends in outcomes between expansion and non-expansion states, which are a check of the difference-in-differences design validity. The pre-trend coefficients are expected to be insignificant (i.e. no differential trends between expansion and non-expansion states prior to the pandemic) if the difference-in-differences assumption that the trends would have evolved similarly between expansion and non-expansion states during the pandemic is valid. When the pre- trends are significant, their signs relative to the post-trends might suggest the direction of bias in the difference-in-differences estimates if those pre-pandemic trends were to continue in the same direction during the pandemic years. When the pre- and post-trend coefficients have the same sign, they indicate that the pre-trends are in the opposite (and not in the same) direction as the post-trends. This is because the pre-trend estimates (2016, 2017, and 2018) are the difference between expansion and non-expansion states in the outcome change from each of those earlier years going “forward” to 2019. In contrast, the post-trend estimates (2020, 2021, 2022, and 2023) are the difference between expansion and non-expansion states in the outcome change from each of those later years going “backward” to 2019 (specifically from 2020 to 2019, 2021 to 2019, and 2022 to 2019).

**X** includes conceptually relevant child and household-level covariates including child’s age (included as a 0/1 indicators for each age in years), sex, race/ ethnicity (coded as non-Hispanic Black, non-Hispanic White, Hispanic, and non-Hispanic other races included as one category because of the small sample size of subgroups), highest education level in the household (less than high school, high school, some college, college degree or higher), parental marital status, household income as a percentage of the FPL, and total number of children living in the household. C includes the following time-varying state-level economic and income support policy indicators: unemployment rate, average income, minimum wage, the earned income tax credit (EITC) rate as a percentage of federal credit (0 for states without an EITC program), and a 0/1 indicator for refundable state EITC (0 for states with non-refundable EITC or no EITC). **S** includes state fixed effects (0/1 indicators), which capture time-invariant differences between states.

The regression model is estimated by linear least squares to provide directly interpretable difference-in-differences estimates and is weighted by sampling probability weights from the NSCH dataset. Standard errors are clustered at the state level. Estimates are considered statistically significant at p < 0.05. All statistical analyses are conducted using Stata/SE version 18.0 (Child and Adolescent Health Measurement Initiative, n.d.).

## Results

### Descriptive Analysis

Tables 1 and 2 show unadjusted descriptive statistics (percents or means) separately for Medicaid expansion and non-expansion states in 2016–2019 and 2020–2023 for outcomes and covariates respectively. Overall, this descriptive analysis shows little difference in outcome changes over time between expansion and non-expansion states. Most healthcare access and utilization measures including having usual place for sick care, a personal doctor/nurse, any medical care utilization, preventive medical visits, preventive dental visits, specialist visits, ED visits, and hospital stays showed some decline during the first year of the pandemic with some showing lower levels beyond first year compared to pre-pandemic levels. There was little change in ratings of children’s health status and parental physical health but there was a decline during the pandemic in parental mental health status.

**Table 2:** Covariate descriptive statistics in 2016–2019 and 2020–2023 by medicaid expansion versus non-expansion status

	2016–2019			2020–2023		
	Expansion states	Non-expansion states	*p-value*	Expansion states	Non-expansion states	*p-value*
Child’s age						
0–5 years old	31.8%	30.4%		30.7%	32.7%	
6–11 years old	35.1%	37.1%		33.1%	33.3%	
12–17 years old	33.1%	32.6%	0.674	36.3%	34.0%	0.1562
Parent age						
	38.1	37.5	0.060	38.6	38.0	0.061
	(8.7)	(8.8)		(8.6)	(8.7)	
Child’s sex						
Male	50.9%	53.0%		52.0%	50.4%	
Female	49.1%	47.0%	0.429	48.0%	49.6%	0.2030
Child’s race and ethnicity						
Non-hispanic white	31.2%	26.1%		29.0%	24.1%	
Non-hispanic black	17.2%	29.4%		17.6%	27.1%	
Hispanic	40.9%	38.8%		42.3%	42.7%	
Non-hispanic other races	10.7%	5.8%	0.000***	11.1%	6.1%	0.000***
Highest level of education in household						
Less than high school	22.1%	20.7%		26.0%	24.1%	
High school	34.9%	38.3%		36.7%	37.2%	
Some college	27.0%	26.5%		22.9%	24.4%	
College and above	16.0%	14.4%	0.2649	14.3%	14.3%	0.5553
Number of children in household						
1	22.3%	21.3%		19.7%	20.9%	
2	33.4%	31.8%		32.5%	32.9%	
3	25.5%	26.6%		26.9%	27.0%	
4 +	18.7%	20.3%	0.2871	20.9%	19.2%	0.6675
Household income as % of FPL						
Household income	83.1	81.9	0.271	83.2	83.2	0.535
	(29.6)	(30.0)		(30.0)	(30.8)	
Parental marital status						
Married	52.0%	47.9%		50.0%	49.8%	
Not married	48.0%	52.1%	0.0157*	50.0%	50.2%	0.7571
State-level variables						
State unemployment rate	4.5	4.1	0.000***	5.7	4.7	0.000***
	(0.7)	(0.7)		(2.2)	(1.7)	
State minimum wage	9.3	7.5	0.000***	11.1	7.7	0.000***
	(1.5)	(0.4)		(2.3)	(1.0)	
State earned income tax credit rate (as % of FPL)	0.3 (0.3)	0.0 (0.1)	0.000***	0.2 (0.2)	0.1 (0.2)	0.000***
State average income	55,527.3	47,379.5	0.000***	66,239.0	57,743.2	0.000***
	(9167.7)	(4101.3)		(10,192.4)	(4993.0)	

All descriptive statistics are weighted by NSCH sampling weights. Means and standard deviations (in parentheses) are given for continuous and 1–5 categorical variables (where 1 indicates the lowest value and 5 the highest), while percents are given for binary outcomes. The table shows the significance of differences in those outcomes between expansion and non-expansion states within each period (based on bivariate chi-square tests for categorical variables and t-tests for continuous variables). Note that those differences in levels within each period do not represent the unadjusted version of the difference-in-differences event study estimates represented below as they only test differences in the levels of those outcomes between expansion and non-expansion states within each period

^*^This table shows descriptive statistics for the sample. Covariate summary statistics are based on the regression sample size for any medical utilization as the outcome (36,768). Unadjusted frequencies for covariates for the pre (2016–19) and post (2020–23) periods are given and sorted by Medicaid expansion and non-expansion status. FPL indicates federal poverty level

^*^P < . 05, ^**^P < . 01, ^***^P < . 001

Overall, for health services access outcomes, there was an increase in the proportion of children who experienced unmet healthcare needs particularly during the first year of the pandemic. There were declines in the proportion of children having a usual place for sick care and children who had a personal doctor/nurse during the pandemic. There were declines in mental health treatment (first two years of the pandemic), child specialist visits, ED visits, hospital stays, and parent mental health status during the pandemic, rates of which were not observed in the years prior to the pandemic.

### Difference-in-Differences Estimates

Table 3 reports the event-study difference-in-differences estimates for the ACA Medicaid expansion effects on children’s healthcare access outcomes. We show both the pre-trend and post-trend estimates. As noted above, the pre-trend and post-trend estimates represent going “forward” and “backward” in time. Therefore, when the pre-trend and post-trend estimates have the same sign, it indicates opposite trends over time.

**Table 3:** Difference-in-differences estimates of the medicaid expansion effects on children’s access to healthcare

Outcome	Sample size	2016	2017	2018	2019	2020	2021	2022	2023
Covered by government assistance plan	39,398	0.05 (0.03) [− 0.02,0.11]	− 0.04 (0.04) [− 0.11,0.04]	− 0.04 (0.02) [− 0.08, 0.00]	Ref	0.01 (0.02) [− 0.04,0.06]	0.02 (0.02) [− 0.02,0.06]	− 0.03 (0.03) [− 0.08,0.03]	0.02 (0.02) [− 0.02,0.07]
Any health insurance coverage	43,266	− 0.03 (0.02) [− 0.08, 0.02]	0.00 (0.02) [− 0.04,0.04]	− 0.01 (0.02) [− 0.05, 0.03]	Ref	0.00 (0.02) [− 0.05,0.04]	0.02 (0.02) [− 0.02,0.06]	− 0.01 (0.02) [− 0.04,0.02]	− 0.01 (0.01) [− 0.04,0.02]
Has usual place for sick care	43,251	− 0.08** (0.03) [− 0.14, − 0.02]	− 0.04 (0.04) [− 0.12,0.04]	− 0.09** (0.03) [− 0.15, − 0.03]	Ref	− 0.04 (0.03) [− 0.09,0.02]	− 0.11*** (0.03) [− 0.16, − 0.05]	− 0.04 (0.03) [− 0.09,0.01]	− 0.05 (0.04) [− 0.12, –0.03]
Personal doctor/nurse	43,206	− 0.01 (0.03) [− 0.08, 0.06]	− 0.02 (0.03) [− 0.08–0.03]	− 0.08* (0.03) [− 0.14, − 0.02]	Ref	− 0.05* (0.03) [− 0.10, 0.00]	− 0.07* (0.03) [− 0.13, 0.00]	− 0.01 (0.03) [− 0.07, 0.05]	0.00 (0.06) [− 0.12, 0.12]
Unmet healthcare needs	43,244	− 0.01 (0.01) [− 0.03, 0.01]	− 0.02 (0.02) [− 0.05, 0.02]	− 0.02 (0.01) [− 0.04, 0.01]	Ref	− 0.02 (0.01) [− 0.04, 0.01]	0.01 (0.02) [− 0.02, 0.05]	0.03 (0.02) [− 0.01, 0.07]	− 0.02 (0.02) [− 0.05, 0.01]

The Table reports estimates of the Medicaid expansion effects year-by-year relative to 2019. The estimates are from a difference-in-differences regression that adjusts for age, sex, race and ethnicity, education level of parents, marital status, household income as a percentage of the federal poverty level, number of children living in the household, state fixed effects, year fixed effects, and state unemployment rate, average income, minimum wage, the earned income tax credit (EITC) rate, and an indicator for refundable state EITC. Estimates are weighted by NSCH sampling weights

^*^P < . 05, ^**^P < . 01, ^***^P < . 001

There were no significant differences during the pandemic between expansion and non-expansion states except for a decline in expansion states in having a usual place for sick care by 11 percentage-points in 2021 and having a personal doctor or nurse by 5–7 percentage-points in 2020–2021 (versus 2019) relative to non-expansion states but no significant differences in 2022 and 2023 compared to 2019. For these two outcomes, there were significant but opposite pre-pandemic trends by expansion status (i.e. an increase in outcomes in expansion versus non-expansion states in at least one period) than during the pandemic. There were no significant pre-pandemic trends for the other access outcomes.

Table 4 presents the event-study difference-in-differences estimates for health services utilization. There were declines in 2021 and 2022 in preventive medical visits by 8 and 4 percentage-points respectively (versus 2019) in expansion versus non-expansion states. In contrast, there was an increase in mental health professional treatments each year in 2021–2023 (versus 2019) in expansion states by 3–5 percentage-points and in non-mental health specialist visits by 3–8 percentage-points compared to non-expansion states. There were also significant and opposite pre-pandemic trends in these three outcomes between expansion and non-expansion states in at least one period before the pandemic (i.e. a decline in outcomes in expansion versus non-expansion states over time). There were no significant pre-post differences between expansion and non-expansion states in any medical care utilization, preventive dental visits, ED visits, and hospital stays.

**Table 4:** Difference-in-differences estimates of the medicaid expansion effects on children’s health services utilization in last 12 months

Outcome	Sample size	2016	2017	2018	2019	2020	2021	2022	2023
Medical care utilization	43,394	− 0.03 (0.03) [− 0.10, 0.04]	0.01 (0.05) [− 0.09, 0.11]	− 0.04 (0.06) [− 0.16, 0.08]	Ref	0.00 (0.03) [− 0.06, 0.07]	− 0.05 (0.03) [− 0.11, 0.00]	− 0.01 (0.04) [− 0.09, 0.07]	0.02 (0.02) [− 0.07, 0.03]
Preventive medical visits	43,101	− 0.07* (0.03) [− 0.13, − 0.02]	− 0.03 (0.04) [− 0.11, 0.04]	− 0.07 (0.05) [− 0.16, 0.02]	Ref	− 0.03 (0.02) [− 0.08, 0.01]	− 0.08** (0.03) [− 0.13, − 0.03]	− 0.04* (0.02) [− 0.09, − 0.00]	− 0.04 (0.02) [− 0.09, 0.00]
Preventive dental visits	37,610	0.00 (0.03) [− 0.06, 0.07]	− 0.03 (0.05) [− 0.14, 0.08]	0.02 (0.04) [− 0.07, 0.11]	Ref	− 0.01 (0.03) [− 0.07, 0.05]	− 0.02 (0.04) [− 0.09, 0.06]	0.02 (0.04) [− 0.05, 0.10]	− 0.00 (0.04) [− 0.08, 0.07]
Mental health professional treatment	43,208	0.03* (0.01) [0.00, 0.06]	0.06** (0.02) [0.02, 0.09]	0.03 (0.02) [− 0.01, 0.07]	Ref	0.05 (0.03) [− 0.00, 0.10]	0.04* (0.02) [0.01, 0.07]	0.03* (0.02) [0.00, 0.07]	0.05* (0.02) [0.01, 0.09]
Non-mental health specialist visit	43,121	0.05* (0.02) [0.01, 0.09]	0.09* (0.04) [0.01, 0.16]	0.07* (0.03) [0.01, 0.12]	Ref	0.04 (0.03) [− 0.02, 0.09]	0.03* (0.02) [0.00, 0.06]	0.06* (0.03) [0.01, 0.11]	0.08** (0.02) [0.03, 0.13]
Emergency dept visits	43,301	0.03 (0.04) [− 0.06, 0.11]	− 0.02 (0.04) [− 0.10, 0.06]	0.00 (0.04) [− 0.09, 0.09]	Ref	− 0.00 (0.03) [− 0.06, 0.05]	− 0.04 (0.03) [− 0.11, 0.03]	0.00 (0.03) [− 0.05, 0.06]	0.01 (0.02) [− 0.04, 0.06]
Hospital stay	34,494	N/A	N/A	0.02 (0.02) [− 0.02, 0.06]	Ref	0.03 (0.02) [− 0.00, 0.06]	0.02 (0.01) [− 0.01, 0.05]	0.03 (0.02) [− 0.00, 0.06]	0.02 (0.01) [− 0.01, 0.05]

The Table reports estimates of the Medicaid expansion effects year-by-year relative to 2019. The estimates are from a difference-in-differences regression that adjusts for age, sex, race and ethnicity, education level of parents, marital status, household income as a percentage of the federal poverty level, number of children living in the household, state fixed effects, year fixed effects, and state unemployment rate, average income, minimum wage, the earned income tax credit (EITC) rate, and an indicator for refundable state EITC. Estimates are weighted by NSCH sampling weights

^*^P < . 05, ^**^P < . 01, ^***^P < . 001

Table 5 presents the event-study difference-in-differences estimates for child health and oral health status outcomes. There was a decline in 2020 and 2023 in children’s health status rating in expansion versus non-expansion states by 0.2–0.1 points (on 1–5-point scale from poor to excellent). There was, however, an opposite trend in this outcome in 2018–2019 (i.e. an outcome improvement prior to the pandemic) between expansion and non-expansion states. There were no significant differences in pandemic or pre-pandemic children’s oral health status between expansion and non-expansion states.

**Table 5:** Difference-in-differences estimates of the medicaid expansion effects on children’s health outcomes

Outcome	Sample Size	2016	2017	2018	2019	2020	2021	2022	2023
General health	43,372	− 0.08 (0.06) [− 0.21, 0.05]	− 0.18 (0.12) [− 0.43, 0.07]	− 0.28*** (0.06) [− 0.41, − 0.15]	Ref	− 0.20*** (0.05) [− 0.31, − 0.10]	− 0.09 (0.07) [− 0.23, 0.06]	− 0.09 (0.06) [− 0.20, 0.03]	− 0.12** (0.04) [− 0.20, − 0.03]
Oral health	42,348	0.06 (0.10) [− 0.14, 0.26]	− 0.11 (0.11) [− 0.33, 0.11]	0.02 (0.11) [− 0.20, 0.25]	Ref	− 0.06 (0.08) [− 0.21, 0.09]	− 0.06 (0.09) [− 0.25, 0.13]	0.03 (0.09) [− 0.16, 0.21]	− 0.04 (0.09) [− 0.23, 0.15]

The Table reports estimates of the Medicaid expansion effects year-by-year relative to 2019. The estimates are from a difference-in-differences regression that adjusts for age, sex, race and ethnicity, education level of parents, marital status, household income as a percentage of the federal poverty level, number of children living in the household, state fixed effects, year fixed effects, and state unemployment rate, average income, minimum wage, the earned income tax credit (EITC) rate, and an indicator for refundable state EITC. Estimates are weighted by NSCH sampling weights

^*^P < . 05, ^**^P < . 01, ^***^P < . 001

Lastly, Table 6 presents the difference-in-differences estimates for parent health outcomes. There was a decline in both physical and mental health status rating for mothers in 2020 in expansion versus non-expansion states by 0.3 and 0.2 points respectively (on 1–5-point scale) but no significant differences in subsequent years. There were also opposite pre-trends in these outcomes before the pandemic. For fathers, there was a decline in mental health in 2020 in expansion versus non-expansion states by 0.2 points but an opposite change (improvement) in both mental and physical health with each improving by 0.2 points in 2023.

**Table 6:** Difference-in-differences estimates of the medicaid expansion effects on parent health outcomes

Outcome	Sample size	2016	2017	2018	2019	2020	2021	2022	2023
Mother’s physical health	33,835	− 0.06 (0.06) [− 0.18, 0.07]	− 0.14** (0.05) [− 0.24, − 0.04]	− 0.29** (0.10) [− 0.49, − 0.09]	Ref	− 0.28*** (0.07) [− 0.42, − 0.13]	0.02 (0.05) [− 0.09, 0.12]	− 0.08 (0.11) [− 0.31, 0.14]	− 0.06 (0.10) [− 0.26, 0.15]
Mother’s mental health	33,834	− 0.04 (0.04) [− 0.12, 0.04]	− 0.18^*^ (0.07) [− 0.33, − 0.03]	− 0.26** (0.08) [− 0.42, − 0.09]	Ref	− 0.21* (0.09) [− 0.39, − 0.03]	0.01 (0.06) [− 0.10, 0.12]	− 0.08 (0.10) [− 0.27, 0.12]	0.05 (0.08) [− 0.11, 0.21]
Father’s physical health	21,002	0.10 (0.11) [− 0.13, 0.32]	0 (0.11) [− 0.23, 0.23]	− 0.08 (0.10) [− 0.29, 0.13]	Ref	0.06 (0.11) [− 0.15, 0.27]	0.07 (0.06) [− 0.06, 0.19]	− 0.03 (0.11) [− 0.26, 0.20]	0.20^*^ (0.09) [0.02, 0.38]
Father’s mental health	21,012	0.08 (0.10) [− 0.13, 0.29]	− 0.09 (0.13) [− 0.35, 0.17]	− 0.09 (0.09) [− 0.28, 0.10]	Ref	− 0.15** (0.06) [− 0.26, − 0.04]	0.03 (0.06) [− 0.10, 0.16]	− 0.10 (0.10) [− 0.30, 0.11]	0.20** (0.07) [0.06, 0.35]

The Table reports estimates of the Medicaid expansion effects year-by-year relative to 2019. The estimates are from a difference-in-differences regression that adjusts for age, sex, race and ethnicity, education level of parents, marital status, household income as a percentage of the federal poverty level, number of children living in the household, state fixed effects, year fixed effects, and state unemployment rate, average income, minimum wage, the earned income tax credit (EITC) rate, and an indicator for refundable state EITC. Estimates are weighted by NSCH sampling weights

^*^P < . 05, ^**^P < . 01, ^***^P < . 001

### Sensitivity Analyses

Tables A2 through A5 report the results from the sensitivity analysis excluding two states that expanded Medicaid in 2016. The results are overall similar to the main analysis except for no significant differences in having a personal doctor or nurse during the pandemic between expansion and non-expansion states (Table A2) and an increase in hospital stays in expansion states in 2020 and 2022 (Table A3).

Tables A6 through A9 report the results from the sensitivity analysis limiting the sample to income up to 100% of the FPL. The results are also generally similar to the main estimates except for no significant pandemic differences in having a personal doctor or nurse (Table A6) and no differences in mother’s mental health in 2020 and father’s mental and physical health in 2023 (Table A9) between expansion and non-expansion states.

## Discussion

This study examines the effects of ACA Medicaid expansions for adults that occurred before the pandemic on children’s healthcare access and utilization, child health status, and parental health status during the pandemic. Employing nationally representative survey data and event-study difference-in-differences analyses, we find heterogeneous effects from these earlier expansions on the evaluated outcomes from 2020 through 2023. Compared to 2019 (pre-pandemic), there is a decline in reporting having a usual place for sick care, a personal doctor or nurse, and preventive medical visits in the first year or two of the pandemic (2021 and/or 2022) in expansion states relative to non-expansion states. Also, there is adecline in children’s health status rating in 2020 and 2023 (versus 2019) in expansion versus non-expansion states. In contrast, there is an increase in the likelihood of mental health professional treatments and other specialist visits in each year in 2021–2023 (versus 2019) in expansion versus non-expansion states. Lastly, there was a decline in maternal physical and mental health and father’s mental health in 2020 in expansion versus non-expansion states, but opposite differences in father’s mental and physical health in 2023.

These heterogeneous effects may be explained, at least in part, by the intertwined effects of prior expansions on improving healthcare access versus other pandemic-related contemporaneous events that differentially affected access and health status between expansion and non-expansion states, namely the extent of social mobility restrictions especially early during the pandemic. Among the study states, Medicaid expansion states had more restrictions on average in social mobility (such as shelter-in-place mandates and closures of schools, restaurants/bars, and gyms) at the beginning of the pandemic than non-expansion states (McCann, 2021; Ruhm, 2024). For example, using data from one study that summarized those restrictions into an index of activity limitations, we found that the Medicaid expansion states included in our study had a mean score of 9.9 compared with 3.7 among non-expansion states, indicating greater activity restrictions in expansion states (Ruhm, 2024). Another resource ranking states from the least restrictions (1) to the most restrictions (50) shows an average ranking of 31.5 and 13.0 for our study expansion and non-expansion states, respectively. Such restrictions may have further reduced utilization of preventive services and disrupted continuity of care (such as having less access to a usual care place or personal health provider) in expansion states during the pandemic. At the same time, restrictions such as stay-at-home and school closure mandates were associated with worsening mental health status, including increased use of mental health services and diagnoses, which may explain the greater utilization of mental health treatments but also the decline in health status in expansion states both for children and parents (Adams-Prassl et al., 2022; Ferwana & Varshney, 2024). The finding that the mental health decline for mothers and fathers was observed only in 2020 is consistent with an effect of social activity restrictions during the the first year of the pandemic and with their subsequent lifting being potentially associated with a reversal of that decline. Research using another data resource found overall non-significant effects of the Medicaid expansions on mental health of parents or childless adults during the first year of the pandemic, although there was some evidence for improvement in mental health for some demographic subgroups (Oyeka & Wehby, 2023). Those findings are broadly consistent with ours, indicating no apparent added benefits to mental health of parents from the prior Medicaid expansions especially early during the pandemic. Lastly, there might also have been positive spillover effects from a larger proportion of Medicaid insured parents in expansion states (boosted further by the Medicaid continuity provision of the FFCRA) that contributed to the increase in mental health treatments and non-mental health specialist visits for children during the pandemic for children in expansion states. Even though the number of Medicaid enrollees increased both in expansion and non-expansion states during the FFCRA Medicaid continuity enactment, in a low-income sample (for example up to 138% FPL), a greater proportion of parents would have benefited from this continuity in expansion versus non-expansion states due to the higher proportion of those eligible for Medicaid. We find no effect from the ACA Medicaid expansion on children’s public insurance coverage during the pandemic, suggesting that effects from the FFCRA are likely operating through parental coverage.

The findings offer some policy-relevant insights. One implication is that potential benefits to children’s care continuity and preventive care use through increased parental coverage among low-income families may dissipate with extended restrictions on accessing routine and preventive care services during a public health crisis such as the COVID-19 pandemic. Moreover, any switching to telehealth services does not appear to have offset those access constraints for this population. Therefore, it is important that any interventions that restrict access to routine and preventive care to address pandemics or other crises carefully weigh the short- and long-term costs and benefits of such restrictions to population health, including children's health. Relatedly, maintaining access for the whole family to mental health services during such crises is important due to elevated needs. As such, interventions to remove barriers to obtaining insurance coverage for both parents and children, thereby improving access to such services and other specialized medical services—such as expanding parental eligibility in non-expansion states or reducing coverage interruptions as done through the FFCRA—become more salient at such times.

The study has strengths including examining a range of access, utilization, and health outcomes from a nationally representative survey over multiple years covering the duration of the COVID-19 pandemic, and an event-study difference-in-differences design that attempts to account for contemporaneous confounding effects. This design addresses time-invariant characteristics that differ between states including healthcare system factors that changed little over this period. Of particular interest among those factors is provider supply, and whether that has changed with the Medicaid expansions. There is no apparent effect of the Medicaid expansion on pediatrician supply or participation in Medicaid, although the expansion has been associated with an increase in the supply of general internal medicine physicians and participation of other primary care providers in Medicaid (Curto & Bhole, 2022; Escarce et al., 2021). Similarly, the design accounts for the effects of other national policies enacted during the pandemic period that may affect the study outcomes such as the cash payments through the economic incentive payments or expansions to the child tax credits to the extent that those have comparable effects between expansion and non-expansion states in this study sample.

The study also has limitations. Outcomes are self-reported which could introduce measurement error. Such errors, however, are unlikely to be correlated with Medicaid expansion status and, therefore, should not bias the difference-in-differences estimates, although they can reduce precision (increase standard errors). Also, as discussed above, states differed in enacting social restrictions which may have moderated the effects of the prior Medicaid expansions on access and utilization. We do not directly evaluate such interactions; examining those interactions in future research is important. Also, we observe differential pre-pandemic trends in the outcomes that also show differences during the pandemic between expansion and non-expansion states. As the Medicaid expansions were enacted before the pandemic, it is possible that the expansion effects varied over time between expansion and non-expansion states which would lead to pre-pandemic trends. Those pre-trends were in the opposite direction to the post-trends as noted above, suggesting reversal during the pandemic. The study does not investigate or explain the causes of those pre-pandemic trend differences and their changes during the pandemic, which is a future research direction. Importantly, the results should be interpreted as differences in trends between expansion and non-expansion states before versus during the pandemic. Another limitation is that the NSCH questionnaire does not inquire further about the types of mental health services and specialist visits received as noted above. Future research with other data sources with detailed service measures would be useful to specifically identify which services differed in use between the two state groups. Lastly, our study does not evaluate potential heterogeneity within this sample, whether by sociodemographic or geographic factors. As the NSCH masks data on metro/non-metro status in seventeen of the study states (AK, AZ, CT, HI, ME, MD, MA, MT, NV, NH, ND, SD, UT, VT, VA, WV, WY) included in our sample due to confidentiality, the study did not evaluate potential heterogeneity in access between rural and urban areas within expansion and non-expansion states.

## Conclusion

During the pandemic, children in low-income households in states that enacted ACA Medicaid expansions for adults prior to the pandemic had a decline in care continuity and preventive visits but an increase in mental health treatments and non-mental health specialist visits when compared with children in non-expansion states. There were also declines in child general health status, maternal physical and mental health status, and father’s mental health status during the first pandemic year (and for children during the last year as well) in expansion states relative to non-expansion states. The findings potentially reflect the intertwined effects of greater parental coverage (and its positive spillover effects on children’s healthcare access and utilization) together with more extensive pandemic-related social mobility restrictions in expansion than non-expansion states that potentially disrupted access to routine and preventive services access and worsened health status to a greater extent in expansion states. When weighing the costs and benefits of public health policies during pandemics or other public health crises, policymakers should carefully cconsider disruptions to accessing routine and preventive care, worsening mental health and increased mental health service needs, and the importance of extending insurance coverage to all family members, both parents and children.

## Supplementary Information

Below is the link to the electronic supplementary material.

Supplementary file1 (DOCX 46 kb)

## Data Availability

The data of this study comes from publicly available datasets from the National Survey of Children’s Health (NSCH) and University of Kentucky Center for Poverty Research National Welfare Data.

## Abbreviations

DD: Difference-in-differences
NSCH: National Survey of Children’s Health
UKCPR: University of Kentucky Center for Poverty Research

## References

[CR1] Adams-Prassl, A., Boneva, T., Golin, M., & Rauh, C. (2022). The impact of the coronavirus lockdown on mental health: Evidence from the United States. *Economic Policy,**37*(109), 139–155. 10.1093/epolic/eiac002

[CR2] Andrade, C., Gillen, M., Molina, J. A., & Wilmarth, M. J. (2022). The social and economic impact of COVID-19 on family functioning and well-being: Where do we go from here? *Journal of Family and Economic Issues,**43*(2), 205–212. 10.1007/s10834-022-09848-x 35669394 10.1007/s10834-022-09848-x PMC9136200

[CR3] Andreyeva, E., Rochford, H. I., & Marthey, D. J. (2025). Examining the impact of ACA Medicaid expansion on insurance coverage, access to care and health of low-income parents. *Health Services Research,**60*(5), Article e14625. 10.1111/1475-6773.14625 40251849 10.1111/1475-6773.14625 PMC12461110

[CR4] Auty, S. G., Aswani, M. S., Wahbi, R. N., & Griffith, K. N. (2023). Changes in health care access by race, income, and Medicaid expansion during the COVID-19 pandemic. *Medical Care,**61*(1), 45–49. 10.1097/MLR.0000000000001788 36477619 10.1097/MLR.0000000000001788 PMC9741953

[CR5] Benitez, J. A., Huang, H., & Johnson, P. L. (2023). The relationship between coronavirus disease 2019 (COVID-19) pandemic-linked job losses and health care access and household financial health in Medicaid expansion and nonexpansion states. *Medical Care,**61*(12), 872–881. 10.1097/MLR.0000000000001933 37801548 10.1097/MLR.0000000000001933

[CR6] Bennett, J. (2021, April 15). More have lost their jobs in US than in EU during COVID-19 downturn. *Pew Research Center*. https://www.pewresearch.org/short-reads/2021/04/15/fewer-jobs-have-been-lost-in-the-eu-than-in-the-u-s-during-the-covid-19-downturn/

[CR7] Bullinger, L. R., Gopalan, M., & Lombardi, C. M. (2023). Impacts of publicly funded health insurance for adults on children’s academic achievement. *Southern Economic Journal,**89*(3), 860–884. 10.1002/soej.12614 38845841 10.1002/soej.12614 PMC11156232

[CR8] Chen, C.Y.-C., Byrne, E., & Vélez, T. (2022). Impact of the 2020 pandemic of COVID-19 on families with school-aged children in the United States: Roles of income level and race. *Journal of Family Issues,**43*(3), 719–740. 10.1177/0192513X21994153 38603084 10.1177/0192513X21994153 PMC7957335

[CR9] Child and Adolescent Health Measurement Initiative. (n.d.). *National Survey of Children’s Health fast facts and frequently asked questions*. Data Resource Center for Child and Adolescent Health. https://www.childhealthdata.org/learn-about-the-nsch/FAQ

[CR10] Choi, N. G., DiNitto, D. M., Marti, C. N., & Choi, B. Y. (2022). Telehealth use among older adults during COVID-19: Associations with sociodemographic and health characteristics, technology device ownership, and technology learning. *Journal of Applied Gerontology,**41*(3), 600–609. 10.1177/07334648211047347 34608821 10.1177/07334648211047347 PMC8847316

[CR11] Conmy, A. B., Peters, C., De Lew, N., & Sommers, B. D. (2023, March 2). *Children’s health coverage trends: Gains in 2020–2022 reverse previous coverage losses* (Issue Brief No. HP-2023–07). *Office of the Assistant Secretary for Planning and Evaluation, U.S. Department of Health and Human Services*.

[CR12] Constantin, J., & Wehby, G. L. (2023). Effects of recent Medicaid expansions on infant mortality by race and ethnicity. *American Journal of Preventive Medicine,**64*(3), 377–384. 10.1016/j.amepre.2022.09.026 36481185 10.1016/j.amepre.2022.09.026

[CR13] Corallo, B., & Moreno, S. (2023, April 4). *Analysis of national trends in Medicaid and CHIP**enrollment during the COVID-19 pandemic*. KFF. https://www.kff.org/coronavirus-covid-19/issue-brief/analysis-of-recent-national-trends-in-medicaid-and-chip-enrollment/

[CR14] Curto, V., & Bhole, M. (2022). Impacts of early ACA Medicaid expansions on physician participation. *Health Services Research,**57*(4), 881–891. 10.1111/1475-6773.13925 34897686 10.1111/1475-6773.13925 PMC9264476

[CR15] Czeisler, M. É., Lane, R. I., Petrosky, E., Wiley, J. F., Christensen, A., Njai, R., Weaver, M. D., Robbins, R., Facer-Childs, E. R., Barger, L. K., Czeisler, C. A., Howard, M. E., & Rajaratnam, S. M. W. (2021). Mental health among parents of children aged <18 years and unpaid caregivers of adults during the COVID-19 pandemic—United States, December 2020 and February–March 2021. MMWR. Morbidity and Mortality Weekly Report, 70(24), 879–887. 10.15585/mmwr.mm7024a3 10.15585/mmwr.mm7024a3 PMC8220951 34138835

[CR16] Czeisler, M. É., Marynak, K., Clarke, K. E. N., Salah, Z., Shakya, I., Thierry, J. M., Ali, N., McMillan, H., Wiley, J. F., Weaver, M. D., Czeisler, C. A., Rajaratnam, S. M. W., & Howard, M. E. (2020). Delay or avoidance of medical care because of COVID-19–related concerns—United States, June 2020. *Morbidity and Mortality Weekly Report,**69*(36), 1250–1257. 10.15585/mmwr.mm6936a4 32915166 10.15585/mmwr.mm6936a4 PMC7499838

[CR17] Dalziel, K., Chua, K.-P., Hua, X., Huang, L., Ryan, A., Freed, G. L., Levy, H., & Ayanian, J. Z. (2026). Association of Medicaid expansion with children’s insurance coverage and healthcare utilization. *Health Affairs Scholar*. 10.1093/haschl/qxaf245 41509066 10.1093/haschl/qxaf245 PMC12778330

[CR18] Dudovitz, R. N., Thomas, K., Shah, M. D., Szilagyi, P. G., Vizueta, N., Vangala, S., Shetgiri, R., & Kapteyn, A. (2022). School-age children’s wellbeing and school-related needs during the COVID-19 pandemic. *Academic Pediatrics,**22*(8), 1368–1374. 10.1016/j.acap.2022.01.015 35124282 10.1016/j.acap.2022.01.015 PMC8813784

[CR19] Ejughemre, U., Lyu, W., & Wehby, G. L. (2024). Effects of the continuous Medicaid coverage provision of the Families First Coronavirus Response Act on postpartum Medicaid coverage, depression symptoms, and birth control use. *Health Services Research,**59*(6), Article e14395. 10.1111/1475-6773.14395 10.1111/1475-6773.14395 PMC12120518 39402851

[CR20] Eliason, E. L. (2020). Adoption of Medicaid expansion is associated with lower maternal mortality. *Women’s Health Issues,**30*(3), 147–152. 10.1016/j.whi.2020.01.005 32111417 10.1016/j.whi.2020.01.005

[CR21] Escarce, J. J., Wozniak, G. D., Tsipas, S., Pane, J. D., Brotherton, S. E., & Yu, H. (2021). Effects of the Affordable Care Act Medicaid expansion on the distribution of new general internists across states. *Medical Care,**59*(7), 653–660. 10.1097/MLR.0000000000001523 33956413 10.1097/MLR.0000000000001523 PMC8191468

[CR22] Ferwana, I., & Varshney, L. R. (2024). The impact of COVID-19 lockdowns on mental health patient populations in the United States. *Scientific Reports,**14*, Article 5689. 10.1038/s41598-024-55879-9 38454064 10.1038/s41598-024-55879-9 PMC10920688

[CR23] Robert Wood Johnson Foundation. (2013, March). *How does employment, or unemployment, affect health? Health Policy Snapshot: Public Health and Prevention*. https://www.rwjf.org/en/insights/our-research/2012/12/how-does-employment--or-unemployment--affect-health-.html

[CR24] Gao, Y. N., & Olfson, M. (2026). Trends in adult outpatient psychotherapy use in the United States, 2019–2023. *Psychiatric Services,**77*(2), 175–178. 10.1176/appi.ps.20250377 41331829 10.1176/appi.ps.20250377 PMC12771505

[CR25] Garfield, R., Orgera, K., & Damico, A. (2019). *The uninsured and the ACA: A primer—Key facts about health insurance and the uninsured amidst changes to the Affordable Care Act*. *KFF*. https://www.kff.org/uninsured/report/the-uninsured-and-the-aca-a-primer-key-facts-about-health-insurance-and-the-uninsured-amidst-changes-to-the-affordable-care-act/

[CR26] Gopalan, M., Lombardi, C. M., & Bullinger, L. R. (2022). Effects of parental public health insurance eligibility on parent and child health outcomes. *Economics & Human Biology,**44*, Article 101098. 10.1016/j.ehb.2021.101098 34929550 10.1016/j.ehb.2021.101098 PMC9301861

[CR27] https://aspe.hhs.gov/sites/default/files/documents/77d7cc41648a371e0b5128f0dec2470e/aspe-childrens-health-coverage.pdf

[CR28] Hudson, J. L., & Moriya, A. S. (2017). Medicaid expansion for adults had measurable “welcome mat” effects on their children. *Health Affairs,**36*(9), 1643–1651. 10.1377/hlthaff.2017.0347 28874493 10.1377/hlthaff.2017.0347

[CR29] Kader, M. R., Rahman, M. M., Bristi, P. D., & Ahmmed, F. (2024). Change in mental health service utilization from pre- to post-COVID-19 period in the United States. *Heliyon,**10*(22), Article e40454. 10.1016/j.heliyon.2024.e40454 39634393 10.1016/j.heliyon.2024.e40454 PMC11616595

[CR30] Kaiser Family Foundation. (2026). Medicaid enrollment and unwinding tracker. https://www.kff.org/medicaid/medicaid-enrollment-and-unwinding-tracker/

[CR31] Lee, H., & Singh, G. K. (2021). Monthly trends in access to care and mental health services by household income level during the COVID-19 pandemic, United States, April–December 2020. *Health Equity,**5*(1), 770–779. 10.1089/heq.2021.0036 34909547 10.1089/heq.2021.0036 PMC8665787

[CR32] Lipton, B. J. (2021). Adult Medicaid benefit generosity and receipt of recommended health services among low-income children: The spillover effects of Medicaid adult dental coverage expansions. *Journal of Health Economics,**75*, Article 102404. 10.1016/j.jhealeco.2020.102404 33291015 10.1016/j.jhealeco.2020.102404

[CR33] Lombardi, C. M., Bullinger, L. R., & Gopalan, M. (2022). Better late than never: Effects of late ACA Medicaid expansions for parents on family health-related financial well-being. *Inquiry: THe Journal of Health Care Organization, Provision, and Financing,**59*, Article 469580221133215. 10.1177/00469580221133215 36354062 10.1177/00469580221133215 PMC9661594

[CR34] Lyu, W., Shane, D. M., & Wehby, G. L. (2020). Effects of the recent Medicaid expansions on dental preventive services and treatments. *Medical Care,**58*(8), 749–755. 10.1097/MLR.0000000000001344 32692142 10.1097/MLR.0000000000001344 PMC8211012

[CR35] Lyu, W., & Wehby, G. L. (2019). The impacts of the ACA Medicaid expansions on cancer screening use by primary care provider supply. *Medical Care,**57*(3), 202–207. 10.1097/MLR.0000000000001053 30624303 10.1097/MLR.0000000000001053

[CR36] Lyu, W., & Wehby, G. L. (2021). Heterogeneous effects of Affordable Care Act Medicaid expansions among women with dependent children by state-level pre-expansion eligibility. *Journal of Women’s Health,**30*(9), 1278–1287. 10.1089/jwh.2020.8776 33555950 10.1089/jwh.2020.8776 PMC8558059

[CR37] Lyu, W., & Wehby, G. L. (2022). Effects of the COVID-19 pandemic on children’s oral health and oral health care use. *Journal of the American Dental Association,**153*(8), 787-796.e2. 10.1016/j.adaj.2022.02.008 35422268 10.1016/j.adaj.2022.02.008 PMC8872823

[CR38] Lyu, W., & Wehby, G. L. (2023a). Changes in children’s health care access and utilization in the United States in the first two years of the COVID-19 pandemic. *Academic Pediatrics,**23*(8), 1462–1470. 10.1016/j.acap.2023.07.007 10.1016/j.acap.2023.07.007 37482298

[CR39] Lyu, W., & Wehby, G. L. (2023b). Child flourishing, school engagement, physical activity, and screen time during the coronavirus disease 2019 pandemic in 2020. *Academic Pediatrics,**23*(3), 659–666. 10.1016/j.acap.2022.12.016 36623586 10.1016/j.acap.2022.12.016 PMC9822554

[CR40] Lyu, W., & Wehby, G. L. (2024). Effects of the Families First Coronavirus Response Act on coverage continuity and access for Medicaid beneficiaries. *Inquiry: THe Journal of Health Care Organization, Provision, and Financing,**61*, Article 469580241282052. 10.1177/00469580241282052 39315678 10.1177/00469580241282052 PMC11425735

[CR41] Lyu, W., & Wehby, G. L. (2026). Effects of continuous Medicaid coverage in 2020–2023 on children’s health insurance coverage, access to care, health services use by type, and health status. *Health Services Research,**61*(1), Article e70034. 10.1111/1475-6773.70034 40887787 10.1111/1475-6773.70034 PMC12857483

[CR42] Margerison, C. E., MacCallum, C. L., Chen, J., Zamani-Hank, Y., & Kaestner, R. (2020). Impacts of Medicaid expansion on health among women of reproductive age. *American Journal of Preventive Medicine,**58*(1), 1–11. 10.1016/j.amepre.2019.08.018 31761513 10.1016/j.amepre.2019.08.019 PMC6925642

[CR43] McCann, A. (2021, April 6). States with the fewest coronavirus restrictions. WalletHub. https://wallethub.com/edu/states-coronavirus-restrictions/73818/

[CR44] Miller, S., Johnson, N., & Wherry, L. R. (2021). Medicaid and mortality: New evidence from linked survey and administrative data. *The Quarterly Journal of Economics,**136*(3), 1783–1829. 10.1093/qje/qjab004

[CR45] Miller, S., & Wherry, L. R. (2019). Four years later: Insurance coverage and access to care continue to diverge between ACA Medicaid expansion and non-expansion states. *AEA Papers and Proceedings,**109*, 327–333. 10.1257/pandp.20191046

[CR46] Mukhopadhyay, S. (2022). The effects of Medicaid expansion on job loss–induced mental distress during the COVID-19 pandemic in the US. *SSM - Population Health,**20*, Article 101279. 10.1016/j.ssmph.2022.101279 36341079 10.1016/j.ssmph.2022.101279 PMC9617676

[CR47] Oyeka, O., & Wehby, G. L. (2023). Effects of the affordable care act medicaid expansions on mental health during the COVID-19 pandemic in 2020–2021. *Inquiry: the Journal of Health Care Organization, Provision, and Financing*. 10.1177/00469580231167338 37052143 10.1177/00469580231166738 PMC10102829

[CR48] Pelletier, J. H., Rakkar, J., Au, A. K., Fuhrman, D., Clark, R. S. B., & Horvat, C. M. (2021). Trends in US pediatric hospital admissions in 2020 compared with the decade before the COVID-19 pandemic. *JAMA Network Open,**4*(2), Article e2037227. 10.1001/jamanetworkopen.2020.37227 33576819 10.1001/jamanetworkopen.2020.37227 PMC7881361

[CR49] Radhakrishnan, L., Leeb, R. T., Bitsko, R. H., Carey, K., Gates, A., Holland, K. M., Hartnett, K. P., Kite-Powell, A., DeVies, J., Smith, A. R., van Santen, K. L., Crossen, S., Sheppard, M., Wotiz, S., Lane, R. I., Njai, R., Johnson, A. G., Winn, A., Kirking, H. L., Anderson, K. N. (2022a). Pediatric emergency department visits associated with mental health conditions before and during the COVID-19 pandemic—United States, January 2019–January 2022. MMWR. Morbidity and Mortality Weekly Report, 71(8), 319–324. 10.15585/mmwr.mm7108e2 10.15585/mmwr.mm7108e2 35202358

[CR50] Radhakrishnan, L., Carey, K., Hartnett, K. P., Kite-Powell, A., Zwald, M., Anderson, K. N., Leeb, R. T., Holland, K. M., Gates, A., DeVies, J., Smith, A. R., van Santen, K. L., Crossen, S., Sheppard, M., Wotiz, S., Johnson, A. G., Winn, A., Kirking, H. L., Lane, R. I. & Adjemian, J. (2022b). Pediatric emergency department visits before and during the COVID-19 pandemic—United States, January 2019–January 2022. MMWR. Morbidity and Mortality Weekly Report, 71(8), 313–318. 10.15585/mmwr.mm7108e1 10.15585/mmwr.mm7108e1 35202351

[CR51] Ruhm, C. J. (2024). US state restrictions and excess COVID-19 pandemic deaths. *JAMA Health Forum,**5*(7), Article e242006. 10.1001/jamahealthforum.2024.2006 39058508 10.1001/jamahealthforum.2024.2006 PMC11282449

[CR52] Semprini, J., Lyu, W., Shane, D. M., & Wehby, G. L. (2022). The effects of ACA Medicaid expansions on health after 5 years. *Medical Care Research and Review,**79*(1), 28–35. 10.1177/1077558720972592 33218289 10.1177/1077558720972592 PMC8218574

[CR53] Simon, K., Soni, A., & Cawley, J. (2017). The impact of health insurance on preventive care and health behaviors: Evidence from the first two years of the ACA Medicaid expansions. *Journal of Policy Analysis and Management,**36*(2), 390–417. 10.1002/pam.21972 28378959 10.1002/pam.21972

[CR54] Sommers, B. D., Buchmueller, T., Decker, S. L., Carey, C., & Kronick, R. (2013). The Affordable Care Act has led to significant gains in health insurance and access to care for young adults. *Health Affairs,**32*(1), 165–174. 10.1377/hlthaff.2012.0552 23255048 10.1377/hlthaff.2012.0552

[CR55] Suarez, G. L., Boone, M. H., Burt, S. A., Shewark, E. A., Mitchell, C., Guzman, P., Lopez-Duran, N. L., Klump, K. L., Monk, C. S., & Hyde, L. W. (2025). Parent mental health before and during the COVID-19 pandemic. *Child Psychiatry & Human Development,**56*(5), 1323–1336. 10.1007/s10578-023-01642-6 38141151 10.1007/s10578-023-01642-6 PMC11234901

[CR56] Teasdale, C. A., Borrell, L. N., Shen, Y., Kimball, S., Zimba, R., Kulkarni, S., Rinke, M. L., Fleary, S. A., & Nash, D. (2022). Missed routine pediatric care and vaccinations in US children during the first year of the COVID-19 pandemic. *Preventive Medicine,**158*, Article 107025. 10.1016/j.ypmed.2022.107025 35318030 10.1016/j.ypmed.2022.107025 PMC8933962

[CR57] U.S. Bureau of Labor Statistics. (2023, May 22). Unemployment in families lower in 2022 than before COVID-19 pandemic. *The Economics Daily*. https://www.bls.gov/opub/ted/2023/unemployment-in-families-lower-in-2022-than-before-covid-19-pandemic.htm

[CR58] Ugwi, P., Lyu, W., & Wehby, G. L. (2019). The effects of the Patient Protection and Affordable Care Act on children’s health coverage. *Medical Care,**57*(2), 115–122. 10.1097/MLR.0000000000001021 30439792 10.1097/MLR.0000000000001021

[CR59] University of Kentucky Center for Poverty Research. (2025, July). *UKCPR national welfare**data, 1980–2023*. Lexington, KY. https://ukcpr.uky.edu/resources/national-welfare-data

[CR60] Vasan, A., Kenyon, C. C., Fiks, A. G., & Venkataramani, A. S. (2023). Continuous eligibility and coverage policies expanded children’s Medicaid enrollment. *Health Affairs,**42*(6), 753–758. 10.1377/hlthaff.2022.01465 37276479 10.1377/hlthaff.2022.01465 PMC11299770

[CR61] Venkataramani, M., Pollack, C. E., & Roberts, E. T. (2017). Spillover effects of adult Medicaid expansions on children’s use of preventive services. *Pediatrics,**140*(6), Article e20170953. 10.1542/peds.2017-0953 29133576 10.1542/peds.2017-0953

[CR62] Wehby, G. L. (2022). The impact of household health insurance coverage gains on children’s achievement in Iowa: Evidence from the ACA. *Health Affairs,**41*(1), 35–43. 10.1377/hlthaff.2021.01222 34982630 10.1377/hlthaff.2021.01222

[CR63] Wehby, G. L., & Lyu, W. (2018). The impact of the ACA Medicaid expansions on health insurance coverage through 2015 and coverage disparities by age, race/ethnicity, and gender. *Health Services Research,**53*(2), 1248–1271. 10.1111/1475-6773.12711 28517042 10.1111/1475-6773.12711 PMC5867173

[CR64] Whaley, C. M., Pera, M. F., Cantor, J., et al. (2020). Changes in health services use among commercially insured U.S. populations during the COVID-19 pandemic. *JAMA Network Open,**3*(11), e2024984. 10.1001/jamanetworkopen.2020.24984 33151319 10.1001/jamanetworkopen.2020.24984 PMC7645698

[CR65] Wherry, L. R., & Miller, S. (2016). Early coverage, access, utilization, and health effects associated with the Affordable Care Act Medicaid expansions. *Annals of Internal Medicine,**164*(12), 795–803. 10.7326/M15-2234 27088438 10.7326/M15-2234 PMC5021068

[CR66] Wright, B., Anderson, D., Whitaker, R., Shrader, P., Bettger, J. P., Wong, C., & Shafer, P. (2021). Comparing health care use and costs among new Medicaid enrollees before and during the COVID-19 pandemic. *BMC Health Services Research,**21*(1), Article 1152. 10.1186/s12913-021-07027-6 34696801 10.1186/s12913-021-07027-6 PMC8544632

[CR67] Zamarro, G., & Prados, M. J. (2021). Gender differences in couples’ division of childcare, work, and mental health during COVID-19. *Review of Economics of the Household,**19*(1), 11–40. 10.1007/s11150-020-09534-7 33488316 10.1007/s11150-020-09534-7 PMC7811157

