# Supplementary material for: Effects of State Medicaid Expansions Affecting Parental Eligibility on Children’s Healthcare Access and Health During the COVID-19 Pandemic
Source: Matern Child Health J. 2026 Aug 7;30(8):1080–93. doi: 10.1007/s10995-026-04292-x (PMC13503441; doi:10.1007/s10995-026-04292-x)
Supplement: Supplementary file 1 [file 10995_2026_4292_MOESM1_ESM.docx]

**APPENDIX**

**Table A1.** Composition of Treatment and Control Group

| **Treatment Group (States which expanded Medicaid by 2016)** | **Control (States which had not expanded Medicaid by 2023** | **Excluded States** |
| --- | --- | --- |
| Alaska | Alabama | Idaho |
| Arizona | Florida | Maine |
| Arkansas | Georgia | Missouri |
| California | Kansas | N. Carolina |
| Colorado | Mississippi | Nebraska |
| Connecticut | S. Carolina | Oklahoma |
| Delaware | Tennessee | S. Dakota |
| DC | Texas | Utah |
| Hawaii | Wisconsin | Virginia |
| Illinois | Wyoming |  |
| Indiana |  |  |
| Iowa |  |  |
| Kentucky |  |  |
| Louisiana |  |  |
| Maryland |  |  |
| Massachusetts |  |  |
| Michigan |  |  |
| Minnesota |  |  |
| Montana |  |  |
| Nevada |  |  |
| New Hampshire |  |  |
| New Jersey |  |  |
| New Mexico |  |  |
| New York |  |  |
| North Dakota |  |  |
| Ohio |  |  |
| Oregon |  |  |
| Pennsylvania |  |  |
| Rhode Island |  |  |
| Vermont |  |  |
| Washington |  |  |
| West Virginia |  |  |
|  |  |  |
|  |  |  |
|  |  |  |

**Table A2:** Difference-in-Differences Estimates of the Medicaid Expansion Effects on Children’s Access to Health Care

Excluding States that Expanded in 2016

| **Outcome** | **Sample Size** | **2016** | **2017** | **2018** | **2019** | **2020** | **2021** | **2022** | **2023** |
| --- | --- | --- | --- | --- | --- | --- | --- | --- | --- |
| Covered by  Government  Assistance Plan | 37,212 | 0.05  (0.03)  [-0.02, 0.11] | -0.04  (0.04)  [-0.12, 0.04] | -0.04  (0.02)  [-0.08, 0.00] | **Ref.** | 0.02  (0.02)  [-0.03, 0.07] | 0.02  (0.02)  [-0.01, 0.06] | -0.02  (0.03)  [-0.08, 0.03] | 0.03  (0.02)  [-0.02, 0.08] |
| Any Health  Insurance  Coverage | 40,872 | -0.03  (0.02)  [-0.08, 0.02] | -0.01  (0.02)  [-0.05, 0.04] | -0.01  (0.02)  [-0.05, 0.03] | **Ref.** | -0.01  (0.02)  [-0.05, 0.04] | 0.01  (0.02)  [-0.03, 0.05] | -0.01  (0.02)  [-0.04, 0.02] | -0.01  (0.01)  [-0.04, 0.02] |
| Has Usual Place  for Sick Care | 40,863 | -0.08**  (0.03)  [-0.14, -0.02] | -0.04  (0.04)  [-0.12, 0.05] | -0.10**  (0.03)  [-0.16, -0.04] | **Ref.** | -0.04  (0.03)  [-0.09, 0.02] | -0.12***  (0.03)  [-0.16, -0.05] | -0.04  (0.03)  [-0.09, 0.01] | -0.05  (0.04)  [-0.13, 0.02] |
| Personal  Doctor/  Nurse | 40,824 | -0.001  (0.03)  [-0.14, -0.02] | -0.02  (0.03)  [-0.12, 0.05] | -0.07*  (0.03)  [-0.16, -0.04] | **Ref.** | -0.05  (0.03)  [-0.09, 0.02] | -0.06  (0.03)  [-0.16, -0.05] | -0.01  (0.03)  [-0.09, 0.01] | -0.001  (0.06)  [-0.13, 0.02] |
| Unmet Healthcare  Needs | 40,854 | -0.02  (0.01)  [-0.04, 0.01] | -0.02  (0.02)  [-0.05, 0.02] | -0.02  (0.02)  [-0.05, 0.01] | **Ref.** | -0.02  (0.01)  [-0.04, 0.01] | 0.01  (0.02)  [-0.03, 0.05] | 0.03  (0.02)  [-0.01, 0.07] | -0.02  (0.02)  [-0.05, 0.01] |

Notes: The Table reports estimates of the Medicaid expansion effects year-by-year relative to 2019. The estimates are from a difference-in-differences regression that adjusts for age, sex, race and ethnicity, education level of parents, marital status, household income as a percentage of the federal poverty level, number of children living in the household, state fixed effects, year fixed effects, and state unemployment rate, average income, minimum wage, the earned income tax credit (EITC) rate, and an indicator for refundable state EITC. Estimates are weighted by NSCH sampling weights.

*	*P* <. 05

**	*P* <. 01

***	*P* <. 001

**Table A3:** Difference-in-Differences Estimates of the Medicaid Expansion Effects on Children’s Health Services Utilization in Last 12 Months Excluding States that Expanded in 2016

| **Outcome** | **Sample Size** | **2016** | **2017** | **2018** | **2019** | **2020** | **2021** | **2022** | **2023** |
| --- | --- | --- | --- | --- | --- | --- | --- | --- | --- |
| Medical Care  Utilization | 40,997 | -0.03  (0.04)  [-0.10, 0.04] | 0.01  (0.05)  [-0.09, 0.11] | -0.04  (0.06)  [-0.16, 0.08] | **Ref.** | 0.00  (0.03)  [-0.06, 0.07] | -0.05  (0.03)  [-0.11, 0.00] | -0.01  (0.04)  [-0.09, 0.08] | -0.02  (0.03)  [-0.08, 0.03] |
| Preventive Medical  Visits | 40,724 | -0.08*  (0.03)  [-0.13, -0.02] | -0.04  (0.04)  [-0.11, 0.04] | -0.07  (0.05)  [-0.16, 0.03] | **Ref.** | -0.03  (0.02)  [-0.08, 0.01] | -0.08**  (0.03)  [-0.14, -0.03] | -0.04*  (0.02)  [-0.09, -0.00] | -0.04  (0.02)  [-0.09, 0.01] |
| Preventive Dental  Visits | 35,507 | -0.003  (0.03)  [-0.07, 0.06] | -0.04  (0.05)  [-0.15, 0.07] | 0.02  (0.05)  [-0.07, 0.11] | **Ref.** | -0.01  (0.03)  [-0.07, 0.05] | -0.02  (0.04)  [-0.10, 0.06] | 0.03  (0.04)  [-0.05, 0.10] | -0.00  (0.04)  [-0.08, 0.07] |
| Mental Health  Professional  Treatment | 40,825 | 0.03*  (0.01)  [0.01, 0.06] | 0.06**  (0.02)  [0.03, 0.10] | 0.03  (0.02)  [-0.01, 0.08] | **Ref.** | 0.05  (0.03)  [-0.00, 0.10] | 0.04*  (0.02)  [0.01, 0.07] | 0.03  (0.02)  [-0.00, 0.07] | 0.05*  (0.02)  [0.01, 0.09] |
| Non-Mental Health  Specialist Visit | 40,733 | 0.05*  (0.02)  [0.01, 0.09] | 0.09*  (0.04)  [0.02, 0.17] | 0.07*  (0.03)  [0.01, 0.12] | **Ref.** | 0.04  (0.03)  [-0.02, 0.10] | 0.03*  (0.02)  [0.00, 0.06] | 0.06*  (0.03)  [0.01, 0.11] | 0.08**  (0.02)  [0.04, 0.13] |
| Emergency Dept.  Visits | 40,909 | 0.03  (0.04)  [-0.05, 0.11] | -0.02  (0.04)  [-0.10, 0.06] | 0.00  (0.04)  [-0.09, 0.09] | **Ref.** | -0.003  (0.03)  [-0.06, 0.05] | -0.04  (0.03)  [-0.11, 0.03] | 0.01  (0.03)  [-0.05, 0.06] | 0.01  (0.03)  [-0.04, 0.06] |
| Hospital Stay | 32,549 | N/A | N/A | 0.03  (0.02)  [-0.01, 0.06] | **Ref.** | 0.03*  (0.02)  [0.00, 0.07] | 0.02  (0.01)  [-0.01, 0.05] | 0.03*  (0.02)  [0.00, 0.07] | 0.02  (0.01)  [-0.01, 0.05] |

Notes: The Table reports estimates of the Medicaid expansion effects year-by-year relative to 2019. The estimates are from a difference-in-differences regression that adjusts for age, sex, race and ethnicity, education level of parents, marital status, household income as a percentage of the federal poverty level, number of children living in the household, state fixed effects, year fixed effects, and state unemployment rate, average income, minimum wage, the earned income tax credit (EITC) rate, and an indicator for refundable state EITC. Estimates are weighted by NSCH sampling weights.

*	*P* <. 05

**	*P* <. 01

***	*P* <. 001

**Table A4:** Difference-in-Differences Estimates of the Medicaid Expansion Effects on Children’s Health Outcomes Excluding States that Expanded in 2016

| **Outcome** | **Sample Size** | **2016** | **2017** | **2018** | **2019** | **2020** | **2021** | **2022** | **2023** |
| --- | --- | --- | --- | --- | --- | --- | --- | --- | --- |
| General Health | 40,976 | -0.09  (0.07)  [-0.22, 0.05] | -0.19  (0.13)  [-0.44, 0.06] | -0.29***  (0.07)  [-0.42, -0.16] | **Ref.** | -0.20***  (0.05)  [-0.31, -0.10] | -0.10  (0.07)  [-0.24, 0.05] | -0.09  (0.06)  [-0.20, 0.03] | -0.12**  (0.04)  [-0.21, -0.04] |
| Oral Health | 40,003 | 0.06  (0.10)  [-0.15, 0.26] | -0.12  (0.11)  [-0.34, 0.12] | 0.02  (0.11)  [-0.21, 0.25] | **Ref.** | -0.06  (0.08)  [-0.21, 0.10] | -0.05  (0.10)  [-0.25, 0.14] | 0.04  (0.09)  [-0.15, 0.22] | -0.03  (0.10)  [-0.22, 0.16] |

Notes: The Table reports estimates of the Medicaid expansion effects year-by-year relative to 2019. The estimates are from a difference-in-differences regression that adjusts for age, sex, race and ethnicity, education level of parents, marital status, household income as a percentage of the federal poverty level, number of children living in the household, state fixed effects, year fixed effects, and state unemployment rate, average income, minimum wage, the earned income tax credit (EITC) rate, and an indicator for refundable state EITC. Estimates are weighted by NSCH sampling weights.

*	*P* <. 05

**	*P* <. 01

***	*P* <. 001

**Table A5:** Difference-in-Differences Estimates of the Medicaid Expansion Effects on Parent Health Outcomes Excluding States that Expanded in 2016

| **Outcome** | **Sample Size** | **2016** | **2017** | **2018** | **2019** | **2020** | **2021** | **2022** | **2023** |
| --- | --- | --- | --- | --- | --- | --- | --- | --- | --- |
| Mother’s Physical Health | 31,993 | -0.06  (0.06)  [-0.19, 0.07] | -0.14*  (0.05)  [-0.24, -0.03] | -0.31**  (0.10)  [-0.51, -0.11] | **Ref.** | -0.28***  (0.07)  [-0.43, -0.13] | 0.01  (0.05)  [-0.10, 0.12] | -0.08  (0.11)  [-0.31, 0.15] | -0.05  (0.10)  [-0.26, 0.16] |
| Mother’s Mental Health | 31994 | -0.05  (0.04)  [-0.13, 0.02] | -0.19^*^  (0.08)  [-0.35, -0.04] | -0.26**  (0.08)  [-0.43, -0.09] | **Ref.** | -0.21*  (0.09)  [-0.39, -0.02] | 0.01  (0.06)  [-0.10, 0.13] | -0.08  (0.10)  [-0.27, 0.12] | 0.06  (0.08)  [-0.10, 0.22] |
| Father’s Physical Health | 20,029 | 0.10  (0.11)  [-0.13, 0.32] | 0  (0.12)  [-0.24, 0.24] | -0.08  (0.11)  [-0.29, 0.13] | **Ref.** | 0.06  (0.11)  [-0.16, 0.28] | 0.07  (0.07)  [-0.06, 0.20] | -0.03  (0.12)  [-0.26, 0.20] | 0.21^*^  (0.09)  [0.03, 0.40] |
| Father’s Mental Health | 20,036 | 0.08  (0.10)  [-0.13, 0.29] | -0.09  (0.13)  [-0.36, 0.17] | -0.10  (0.09)  [-0.29, 0.09] | **Ref.** | -0.15*  (0.06)  [-0.26, -0.03] | 0.04  (0.06)  [-0.09, 0.17] | -0.09  (-0.10)  [-0.30, 0.12] | 0.22**  (0.07)  [0.07, 0.36] |

Notes: The Table reports estimates of the Medicaid expansion effects year-by-year relative to 2019. The estimates are from a difference-in-differences regression that adjusts for age, sex, race and ethnicity, education level of parents, marital status, household income as a percentage of the federal poverty level, number of children living in the household, state fixed effects, year fixed effects, and state unemployment rate, average income, minimum wage, the earned income tax credit (EITC) rate, and an indicator for refundable state EITC. Estimates are weighted by NSCH sampling weights.

*	*P* <. 05

**	*P* <. 01

***	*P* <. 001

**Table A6:** Difference in Differences Estimates for Children’s Access to Health Care in Medicaid Expansion States Limiting the Sample to <= 100% of FPL

| **Outcome** | **Sample Size** | **2016** | **2017** | **2018** | **2019** | **2020** | **2021** | **2022** | **2023** |
| --- | --- | --- | --- | --- | --- | --- | --- | --- | --- |
| Covered by  Government  Assistance Plan | 25,329 | 0.03  (0.03)  [-0.03, 0.10] | -0.01  (0.04)  [-0.09, 0.07] | -0.06*  (0.02)  [-0.10, -0.01] | **Ref.** | 0.01  (0.02)  [-0.03, 0.06] | 0.02  (0.02)  [-0.02, 0.06] | -0.02  (0.03)  [-0.08, 0.04] | 0.03  (0.03)  [-0.03, 0.09] |
| Any Health  Insurance  Coverage | 27,974 | -0.01  (0.03)  [-0.07, 0.04] | -0.03  (0.03)  [-0.10, 0.04] | -0.04  (0.04)  [-0.12, 0.03] | **Ref.** | -0.01  (0.03)  [-0.07, 0.06] | 0.01  (0.02)  [-0.03, 0.04] | -0.02  (0.03)  [-0.07, 0.03] | -0.002  (0.01)  [-0.03, 0.02] |
| Has Usual Place  for Sick Care | 27,951 | -0.10*  (0.04)  [-0.18, -0.01] | -0.04  (0.04)  [-0.11, 0.03] | -0.16***  (0.04)  [-0.23, -0.09] | **Ref.** | -0.06  (0.05)  [-0.16, 0.05] | -0.10**  (0.03)  [-0.16, -0.03] | -0.07  (0.04)  [-0.14, 0.01] | -0.05  (0.04)  [-0.12, 0.03] |
| Personal  Doctor/  Nurse | 27,939 | -0.04  (0.02)  [-0.09, 0.01] | -0.04  (0.04)  [-0.13, 0.05] | -0.12  (0.06)  [-0.24, 0.00] | **Ref.** | -0.03  (0.03)  [-0.09, 0.02] | -0.05  (0.04)  [-0.13, 0.02] | 0.02  (0.04)  [-0.06, 0.09] | 0.00  (0.05)  [-0.09, 0.09] |
| Unmet Healthcare  Needs | 27,946 | -0.02  (0.02)  [-0.05, 0.01] | -0.05  (0.03)  [-0.11, 0.01] | -0.02  (0.02)  [-0.06, 0.02] | **Ref.** | 0.00  (0.02)  [-0.04, 0.04] | 0.01  (0.02)  [-0.03, 0.06] | 0.02  (0.02)  [-0.03, 0.07] | -0.03  (0.02)  [-0.07, 0.00] |

Notes: The Table reports estimates of the Medicaid expansion effects year-by-year relative to 2019. The estimates are from a difference-in-differences regression that adjusts for age, sex, race and ethnicity, education level of parents, marital status, household income as a percentage of the federal poverty level, number of children living in the household, state fixed effects, year fixed effects, and state unemployment rate, average income, minimum wage, the earned income tax credit (EITC) rate, and an indicator for refundable state EITC. Estimates are weighted by NSCH sampling weights.

*	*P* <. 05

**	*P* <. 01

***	*P* <. 001

**Table A7:** Difference in Differences Estimates for Health Services Utilization in Last 12 Months for Children in Medicaid Expansion States Limiting the Sample to <= 100% of FPL

| **Outcome** | **Sample Size** | **2016** | **2017** | **2018** | **2019** | **2020** | **2021** | **2022** | **2023** |
| --- | --- | --- | --- | --- | --- | --- | --- | --- | --- |
| Medical Care  Utilization | 28,053 | -0.04  (0.03)  [-0.10, 0.03] | 0.05  (0.06)  [-0.07, 0.17] | -0.03  (0.04)  [-0.12, 0.05] | **Ref.** | -0.01  (0.03)  [-0.06, 0.04] | -0.04  (0.03)  [-0.09, 0.02] | -0.02  (0.03)  [-0.08, 0.04] | -0.02  (0.03)  [-0.08, 0.05] |
| Preventive Medical  Visits | 27,870 | -0.09*  (0.04)  [-0.17, -0.01] | 0.03  (0.05)  [-0.07, 0.12] | -0.06  (0.04)  [-0.13, 0.01] | **Ref.** | -0.04  (0.02)  [-0.08, 0.00] | -0.07  (0.04)  [-0.14, 0.00] | -0.07**  (0.02)  [-0.12, -0.03] | -0.05  (0.03)  [-0.11, 0.01] |
| Preventive Dental  Visits | 24,192 | -0.02  (0.04)  [-0.10, 0.07] | -0.05  (0.06)  [-0.16, 0.06] | 0.02  (0.07)  [-0.12, 0.15] | **Ref.** | -0.03  (0.04)  [-0.10, 0.04] | -0.01  (0.04)  [-0.10, 0.08] | -0.01  (0.05)  [-0.10, 0.09] | 0.02  (0.05)  [-0.09, 0.12] |
| Mental Health  Professional  Treatment | 27,924 | 0.04**  (0.02)  [0.01, 0.08] | 0.09***  (0.02)  [0.05, 0.13] | 0.02  (0.02)  [-0.03, 0.07] | **Ref.** | 0.06  (0.03)  [-0.01, 0.12] | 0.03  (0.02)  [-0.01, 0.08] | 0.05**  (0.01)  [0.02, 0.07] | 0.04*  (0.02)  [0.00, 0.07] |
| Non-Mental Health  Specialist Visit | 27,876 | 0.05*  (0.02)  [0.01, 0.10] | 0.13**  (0.05)  [0.04, 0.22] | 0.07*  (0.03)  [0.01, 0.13] | **Ref.** | 0.06*  (0.03)  [0.00, 0.12] | 0.03  (0.02)  [-0.01, 0.08] | 0.08*  (0.04)  [0.00, 0.16] | 0.09**  (0.03)  [0.02, 0.16] |
| Emergency Dept.  Visits | 27,988 | 0.04  (0.05)  [-0.06, 0.15] | 0.01  (0.03)  [-0.06, 0.08] | 0.04  (0.06)  [-0.07, 0.15] | **Ref.** | 0.03  (0.05)  [-0.08, 0.13] | 0.02  (0.05)  [-0.09, 0.12] | 0.04  (0.05)  [-0.07, 0.14] | 0.04  (0.05)  [-0.07, 0.15] |
| Hospital Stay | 22,356 | N/A | N/A | 0.03  (0.03)  [-0.02, 0.09] | **Ref.** | 0.04  (0.02)  [-0.01, 0.08] | 0.03  (0.03)  [-0.01, 0.07] | 0.04  (0.02)  [0.00, 0.08] | 0.04  (0.02)  [-0.01, 0.08] |

Notes: The Table reports estimates of the Medicaid expansion effects year-by-year relative to 2019. The estimates are from a difference-in-differences regression that adjusts for age, sex, race and ethnicity, education level of parents, marital status, household income as a percentage of the federal poverty level, number of children living in the household, state fixed effects, year fixed effects, and state unemployment rate, average income, minimum wage, the earned income tax credit (EITC) rate, and an indicator for refundable state EITC. Estimates are weighted by NSCH sampling weights.

*	*P* <. 05

**	*P* <. 01

***	*P* <. 001

**Table A8:** Difference in Differences Estimates for Child Health Outcomes in Medicaid Expansion States Limiting the Sample to <= 100% of FPL

| **Outcome** | **Sample Size** | **2016** | **2017** | **2018** | **2019** | **2020** | **2021** | **2022** | **2023** |
| --- | --- | --- | --- | --- | --- | --- | --- | --- | --- |
| General Health | 28,043 | -0.13  (0.09)  [-0.30, 0.05] | -0.29*  (0.12)  [-0.54, -0.04] | -0.32***  (0.07)  [-0.47, -0.18] | **Ref.** | -0.27***  (0.07)  [-0.41, -0.12] | -0.17*  (0.08)  [-0.32, -0.01] | -0.132  (0.07)  [-0.27, 0.01] | -0.16***  (0.04)  [-0.25, -0.07] |
| Oral Health | 27,354 | 0.14  (0.11)  [-0.08, 0.36] | -0.14  (0.14)  [-0.42, 0.13] | 0.11  (0.12)  [-0.14, 0.36] | **Ref.** | -0.08  (0.05)  [-0.17, 0.02] | -0.05  (0.08)  [-0.22, 0.11] | 0.05  (0.08)  [-0.11, 0.21] | -0.06  (0.07)  [-0.20, 0.08] |

Notes: The Table reports estimates of the Medicaid expansion effects year-by-year relative to 2019. The estimates are from a difference-in-differences regression that adjusts for age, sex, race and ethnicity, education level of parents, marital status, household income as a percentage of the federal poverty level, number of children living in the household, state fixed effects, year fixed effects, and state unemployment rate, average income, minimum wage, the earned income tax credit (EITC) rate, and an indicator for refundable state EITC. Estimates are weighted by NSCH sampling weights.

*	*P* <. 05

**	*P* <. 01

***	*P* <. 001

**Table A9:** Difference in Differences Estimates for Parent Health Outcomes in Medicaid Expansion States Limiting the Sample limited to <= 100% of FPL

| **Outcome** | **Sample Size** | **2016** | **2017** | **2018** | **2019** | **2020** | **2021** | **2022** | **2023** |
| --- | --- | --- | --- | --- | --- | --- | --- | --- | --- |
| Mother’s Physical Health | 21,698 | -0.06  (0.13)  [-0.32, 0.21] | -0.13  (0.06)  [-0.26, -0.01] | -0.30**  (0.11)  [-0.52, -0.08] | **Ref.** | -0.28**  (0.09)  [-0.47, -0.10] | 0.04  (0.05)  [-0.10, 0.12] | -0.06  (0.11)  [-0.31, 0.15] | -0.01  (0.10)  [-0.26, 0.16] |
| Mother’s Mental Health | 21,694 | -0.09  (0.04)  [-0.13, 0.02] | -0.14  (0.08)  [-0.35, -0.04] | -0.21*  (0.08)  [-0.42, -0.09] | **Ref.** | -0.08  (0.09)  [-0.40, -0.02] | -0.01  (0.06)  [-0.10, 0.13] | -0.08  (0.10)  [-0.27, 0.12] | -0.03  (0.08)  [-0.10, 0.22] |
| Father’s Physical Health | 12,601 | 0.02  (0.11)  [-0.125, 0.32] | -0.07  (0.12)  [-0.24, 0.24] | -0.31*  (0.11)  [-0.29, 0.13] | **Ref.** | -0.02  (0.11)  [-0.16, 0.28] | 0.00  (0.07)  [-0.06, 0.20] | -0.21  (0.12)  [-0.26, 0.20] | 0.105  (0.09)  [0.03, 0.40] |
| Father’s Mental Health | 12,609 | -0.03  (0.10)  [-0.13, 0.29] | -0.18  (0.13)  [-0.36, 0.17] | -0.32*  (0.09)  [-0.29, 0.09] | **Ref.** | -0.19*  (0.06)  [-0.26, -0.03] | 0.02  (0.06)  [-0.09, 0.17] | -0.21  (-0.10)  [-0.30, 0.12] | 0.07  (0.07)  [0.07, 0.36] |

Notes: The Table reports estimates of the Medicaid expansion effects year-by-year relative to 2019. The estimates are from a difference-in-differences regression that adjusts for age, sex, race and ethnicity, education level of parents, marital status, household income as a percentage of the federal poverty level, number of children living in the household, state fixed effects, year fixed effects, and state unemployment rate, average income, minimum wage, the earned income tax credit (EITC) rate, and an indicator for refundable state EITC. Estimates are weighted by NSCH sampling weights.

*	*P* <. 05

**	*P* <. 01

***	*P* <. 001
